# HPA-Axis Activity and Nutritional Status Correlation in Individuals with Alcohol Use Disorder

**DOI:** 10.3390/nu14234978

**Published:** 2022-11-23

**Authors:** Kalliopi Georgakouli, Eirini Manthou, Ioannis G. Fatouros, Chariklia K. Deli, Yiannis Koutedakis, Yannis Theodorakis, Athanasios Z. Jamurtas

**Affiliations:** 1 Department of Nutrition and Dietetics, University of Thessaly, 42132 Trikala, Greece; 2 Department of Physical Education and Sport Science, University of Thessaly, 42100 Trikala, Greece

**Keywords:** alcohol, nutrient, body composition, hormone, fat, brain, metabolism

## Abstract

Impaired activity of the hypothalamic–pituitary–adrenal axis (HPA-axis) is evident in alcohol use disorder (AUD), and may be implicated in various nutritional and metabolic alterations often seen in individuals with this disorder. The present study examined a possible correlation between HPA-axis activity and nutritional status components in individuals with AUD. Fourteen AUD and fourteen non-AUD males participated; anthropometric and body composition measurements were made, and fasting blood samples were analysed for plasma adrenal corticotropic hormone (ACTH), catecholamines, cortisol and beta-endorphin. Nutrient intake was estimated via a three-day diet record. Waist circumference and waist-to-hip ratio were increased in the AUD group. Thiamine and folic intake were lower in AUD group, although only folic acid intake was insufficient in both AUD and non-AUD groups. Increased epinephrine and norepinephrine were also observed in AUD group compared to non-AUD group. No clear correlation between HPA-axis activity and nutritional status components was found. This study showed that nutrient intake, body composition, and HPA-axis activity were different among AUD and non-AUD individuals. More research on the correlation between nutritional status and HPA-axis activity in AUD individuals should be conducted.

## 1. Introduction

Acute alcohol intake influences the activity of the hypothalamic–pituitary–adrenal axis (HPA-axis), a major neuroendocrine system, resulting in increased adrenocorticotropin hormone (ACTH) release and, consequently, the production of glucocorticoid hormones (i.e., cortisol). Glucocorticoids are important for the release of dopamine in certain brain areas linked to the reinforcing and rewarding effects of alcohol and other drugs [1]. However, chronic excessive exposure to alcohol may impair HPA-axis activity [2,3], as evidenced by blunted cortisol response to various of physical and psychological stressors [4,5]. These alterations in HPA-axis activity may be accountable for the feelings of discomfort and negative reinforcement [6,7] observed in individuals with alcohol use disorders (AUD), with alcohol serving as a relief from a negative emotional state [8].

AUD is a medical condition that affects the ability to control alcohol use, resulting in social, occupational, or health problems [9]. In addition, malnutrition (an imbalance in energy and nutrient intake) in individuals with AUD is a common finding in the research literature that can cause fatigue, muscle weakness, increased risk of infection and/or associated diseases, as well as symptoms of low mood [10]. The HPA-axis may play a role in nutrient deficiencies and associated metabolic alterations in these individuals [11]. Thus, it has been proposed that a synergistic effect of addictive substances (including alcohol) and nutritional status may occur, with addictive substances dysregulating the release of gastrointestinal hormones involved in the regulation of the sensations of appetite and satiety [12]. Consequently, all these changes result in alterations in body weight and/or composition, which in turn often result in metabolic disorders and diseases over time. Moreover, it should also be taken into consideration that nutrition deficiencies can compromise optimal brain functioning, contributing to mood changes [13]. Together, the association of alcohol misuse and malnutrition could be seen as a vicious circle and HPA-axis could be the link.

The aim of this investigation was (a) to compare HPA-axis activity and nutritional status in individuals with and without AUD (b) to examine whether a correlation between HPA-axis activity and nutritional status in individuals with AUD exists. Specifically, the relation of catecholamines and other HPA-axis related hormones were assessed, and their relationship with body composition and dietary data was examined.

## 2. Materials and Methods

### 2.1. Participants

Recruitment of volunteers was conducted in the region of Thessaly, Greece through paper announcements, social media, and face-to-face communications. A physician took the medical history and performed a physical examination of each volunteer. Eligible volunteers were informed about all the aspects of the study, and signed an informed consent document.

Inclusion criteria: only male gender participants; age group 18–60 years.

Exclusion criteria: high physical activity level; major health problems; current or previous substance abuse (except from alcohol).

All participants exhibited low to moderate physical activity, as estimated by the International Physical Activity Questionnaire (IPAQ-Gr) [14]. Regarding alcohol intake, participants in the AUD group exceeded the drinking levels defined by the National Institute on Alcohol Abuse and Alcoholism [15]. In addition, in order to identify those will an AUD, participants completed the Alcohol Use Disorders Identification Test (AUDIT, [16]), a method for screening risky drinking [17]. AUDIT contains 10 items (each answer gives a score from 0 to 4) about alcohol use, symptoms of dependence, and associated issues [17]. Participants with AUDIT score ≥ 8 were assigned to the AUD group (n = 14), while participants with AUDIT score < 8 were assigned to the non-AUD group (n = 14). Scores from 8 to 15 indicate hazardous drinking, which increases the chance of adverse effects [18]; scores from 16 to 19 indicate harmful drinking, which causes health harm and possibly social consequences [18]; scores of ≥20 indicate serious abuse/dependence [17]. Except for physical activity level, groups were matched for age and gender (males) to avoid differences in body composition and other physiological parameters that exist between genders and could make analyses more difficult.

### 2.2. Experimental Design

Ethical approval was received from the Internal Ethics Committee of the University of Thessaly. The methods were in consistent with the Declaration of Helsinki (1975).

Participants were tutored to complete a three-day diet record. They reported to the laboratory between 8:00 and 9:00 a.m. (after refraining from food and smoking overnight) for the measurement of anthropometric and physiological parameters and a blood sample collection for later analysis of biochemical indices.

### 2.3. Anthropometric and Body Composition Measurements

Participants wore light clothes and no shoes for the measurement of body weight with a precision of 100 g and body composition (Tanita TBF−521 Body Monitor/Scale; Tanita Corporation of America Inc., IL, USA), and for the measurement of standing height with a precision of 1 mm (Stadiometer 208; Seca, Birmingham, UK). Then, body mass index (BMI) was computed by the equation: [body mass (in kg)]/[squared body height (in m)]. Waist and hip circumferences were measured according to ACSM’s Guidelines [19]. Waist to hip ratio (WHR) was also calculated as follows: [waist circumference (in cm)]/[hip circumference (in cm)].

### 2.4. Dietary Analysis

A registered dietitian instructed the participants on how to complete a three-day diet record in detail (including one weekend day). The three-day diet records were analysed by the Science Fit Diet 200 A (Science Technologies, Athens, Greece) for the evaluation of the mean daily intake of energy, nutrients, and alcohol.

### 2.5. Blood Collection and Handling

Participants were asked to maintain their usual habits regarding food and physical activity for three days prior to blood sampling

Blood samples were obtained from a forearm vein, taking all the precautions needed. The preparation of plasma for the determination of beta-endorphin, catecholamines, and adrenocorticotropic hormone (ACTH), and serum for the determination of cortisol were previously described [20].

### 2.6. Assays

Each parameter was analysed twice and on the same day, and each sample underwent a single freeze-thaw cycle. The assays and kits used for the determination of the concentrations of the biochemical indices were previously described as well [20].

### 2.7. Statistical Analysis

Descriptive statistics were calculated for the characteristics of the tested groups, with results expressed as mean ± standard deviation (SD). Normality of the tested variables was assessed by a Shapiro–Wilk test (n = 14 per group). It was shown that most variables differed significantly from normal distribution and non-parametric methods were applied. The Mann–Whitney U test was used to investigate differences in the tested variables between groups (AUD vs non-AUD individuals). The median of the variables was also calculated in order to report the results of Mann–Whitney U test. Finally, a Pearson correlation coefficient was calculated for each group. Then, to compare correlation coefficients between the tested groups, group-wise correlation analysis was performed and Fisher z-transformation score was calculated. The level of statistical significance was set at *p* < 0.05. The statistical software used was IBM SPSS Version 19.0 (IBM Corp., Armonk, NY, USA).

## 3. Results

### 3.1. Descriptive Statistics

Table 1 presents the characteristics of the participants in each group in terms of age, physical activity level, alcohol use and smoking habits. AUD group had an AUDIT score > 15 and mean alcohol intake of 17.9 standard drinks per week (one standard drink equivalent is 14 g of alcohol) that corresponds to 35.8 g per day; whereas non-AUD group had an AUDIT score < 8 and mean alcohol intake of 2.9 standard drinks per week that corresponds to 5.8 g per day.

### 3.2. Group Comparisons

The results in Table 2 show that the differences were statistically significant for WC (AUD: 98.1 ± 11.4, 95% CI [91.6, 104.7] vs non-AUD: 86.8±5.6, 95% CI [83.6, 90.0]) and WHR (AUD: 0.94 ± 0.10, 95% CI [0.89, 1.00] vs non-AUD: 0.86±0.01, 95% CI [0.84, 0.88]); the World Health Organization defines abdominal adiposity in men as a WHR of 0.90 or more) but not for BMI (AUD: 28.1 ± 2.8, 95% CI [26.5, 29.7] vs non-AUD: 27.7 ± 3.9, 95% CI [25.4, 29.9]) and body fat (BF) (AUD: 21.8 ± 4.7, 95% CI [19.1, 24.5] vs non-AUD: 22.2 ± 4.3, 95% CI [19.7, 24.7]); BF for the average male population (30−50 yrs) is from 11 to 17% according to Jeukendrup and Gleeson, 2010 [21])], verifying that AUD individuals have abnormal fat distribution (abdominal adiposity).

Regarding macronutrients, in the AUD group carbohydrates supplied 31.6%, proteins 13%, fats 33.6%, and alcohol 21.8% of the total energy (caloric) intake. In the non-AUD group carbohydrates supplied 42.4%, proteins 16.4%, fats 35.7%, and alcohol 5.5% of the total caloric intake. A Mann–Whitney U test showed that there was a significant difference (U = 6000, Z=−4.231; *p* < 0.001) in alcohol intake between the AUD and non-AUD group. The median alcohol intake was 48.82 g for the AUD group compared to 14 g for the non-AUD group (AUD: 53.9 ± 29.3, 95% CI [37.0, 70.1] vs non-AUD: 12.2 ± 5.9, 95% CI [8.8, 15.6]) suggesting that AUD group’s alcohol intake exceeded the normal limit (i.e., up to 20 g per day).

Regarding micronutrients, a significant difference (U = 40000, Z = −2.666; *p* = 0.007) in Fe intake between the AUD and non-AUD group was shown (Table 3). The median Fe intake was 10.265 mg for the AUD group compared to 16.65 mg for the non-AUD group (AUD: 10.6 ± 4.6, 95% CI [8.0, 13.2] vs non-AUD: 16.3 ± 4.6, 95% CI [13.7, 18.9]); however, both groups had sufficient Fe intake (recommended dietary allowance—RDA: 8 mg). A significant difference (U = 47000, Z = −2.344; *p* = 0.019) in B1 intake between the AUD and non-AUD group was shown (Table 3). The median B1 intake was 1.35 mg for the AUD group compared to 2.1 mg for the non-AUD group (AUD: 1.43±0.70, 95% CI [1.03, 1.83] vs non-AUD: 2.42 ± 1.18, 95% CI [1.74, 3.09]); however, both groups had sufficient B1 intake (RDA: 1.2). Finally, a significant difference (U = 47000, Z = −2.343; *p* = 0.019) in folic acid intake between the AUD and non-AUD group was shown (Table 3). The median folic acid intake was 240.65 μg for the AUD group compared to 354.15 μg for the non-AUD group (AUD: 256.2 ± 138.0, 95% CI [176.5, 335.9] vs non-AUD: 368.0 ± 113.0, 95% CI [302.8, 433.2]); however, both groups had insufficient folic acid intake (RDA: 400).

A Mann–Whitney U test showed that there was a significant difference (U = 16000, Z = −3.771; *p* < 0.001) in beta-endorphin concentrations between the AUD and non-AUD group (Table 4). The median beta-endorphin concentrations were 3.35 pg/mL for the AUD group compared to 8.1 pg/mL for the non-AUD group (AUD: 3.89 ± 2.14, 95% CI [2.69, 5.12] vs. non-AUD: 7.69 ± 1.71, 95% CI [6.70, 8.67]).

A significant difference (U = 52000, Z = −2.120; *p* < 0.05) in epinephrine concentrations between the AUD and non-AUD group was shown (Table 4). The median epinephrine concentrations were 37.5 pg/mL for the AUD group compared to 32.0 pg/mL for the non-AUD group (AUD: 42.2 ± 10.8, 95% CI [35.9, 48.5] vs. non-AUD: 31.5 ± 8.7, 95% CI [26.5, 36.5]; normal limit: <100 pg/mL).

Finally, a significant difference (U = 42500, Z = −2.551; *p* < 0.05) in norepinephrine concentrations between the AUD and non-AUD group was shown (Table 4). The median norepinephrine concentrations were 289.5 pg/mL for the AUD group compared to 194.5 pg/mL for the non-AUD group (AUD: 309.7 ± 128.6, 95% CI [235.5, 383.9] vs non-AUD: 196.1 ± 36.3, 95% CI [175.1, 217.0]; normal limit: <600 pg/mL).

### 3.3. Correlation between HPA-Axis Activity and Nutritional Status

**Figure 1:**
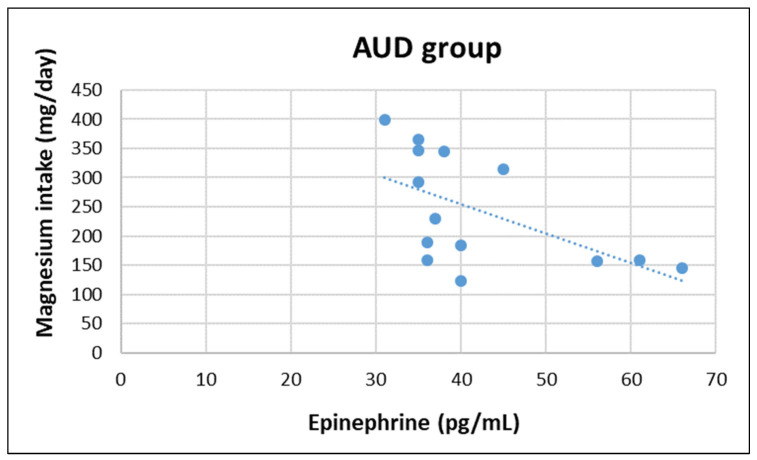
Correlation between plasma epinephrine concentrations and daily magnesium intake in AUD group.

**Figure 2:**
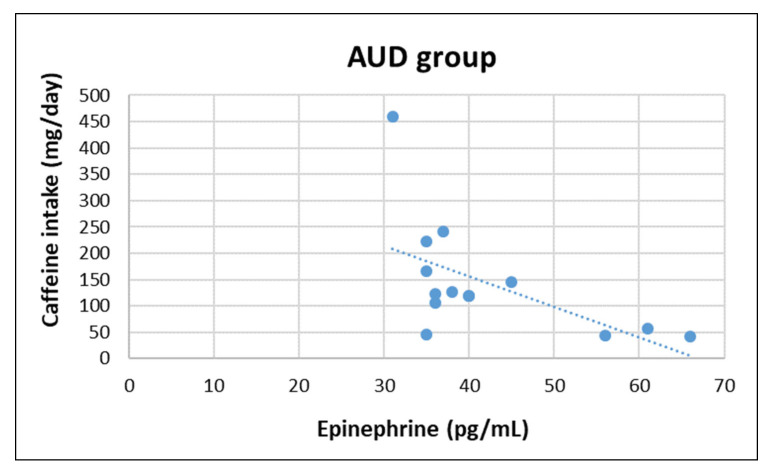
Correlation between plasma epinephrine concentrations and daily caffeine intake in AUD group.

**Figure 3:**
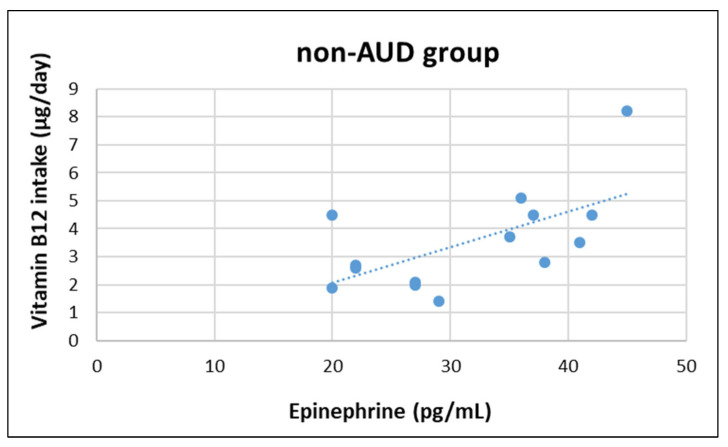
Correlation between plasma epinephrine concentrations and daily vitamin B12 intake in non-AUD group.

**Figure 4:**
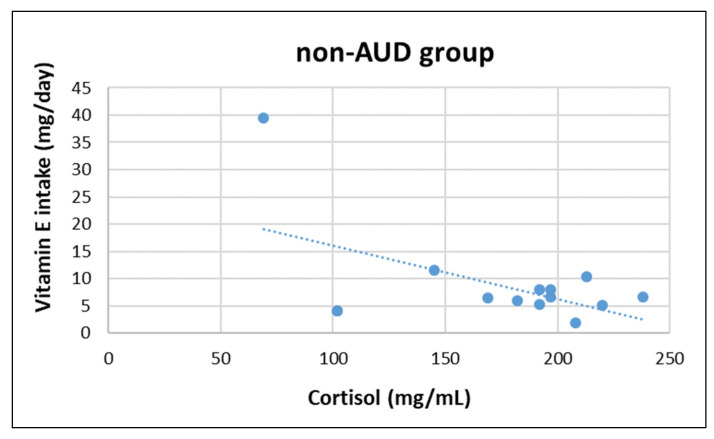
Correlation between plasma cortisol concentrations and daily vitamin E intake in non-AUD group.

In AUD group, epinephrine concentration was negatively correlated with magnesium (z = −2.252, r = −570, *p* = 0.033) and caffeine intake (z = −2.100, r = −577, *p* = 0.031), whereas in non-AUD group epinephrine concentration was positively correlated with vitamin B12 (z = 2.479, r = 0.621, *p* = 0.018). Moreover, cortisol concentration was negatively correlated with vitamin E (z = −2.192, r = −0.543, *p* = 0.045) in non-AUD group (see Figure 1, Figure 2, Figure 3 and Figure 4). No other significant correlation was detected.

## 4. Discussion

This study showed that nutrient intake, body composition, and HPA-axis activity differed among AUD and non-AUD individuals. More research on the correlation between nutritional status and HPA-axis activity in AUD individuals should be conducted.

Abdominal adiposity was evident only in the AUD group (as indicated by increased waist circumference and WHR), and alcohol may have played a role [22]. The AUD and non-AUD group were matched for age and body mass index, with individuals in both groups being overweight (as indicated by BMI and BF); however, only AUD individuals had increased WHR. Caloric intake, caloric expenditure, and macronutrient distribution (percentage of total caloric intake) were similar between groups, whereas the contribution of alcohol intake to total caloric intake was different. Specifically, the AUD group had increased alcohol intake (21.8% vs. 5.5%) and non-significant decreased carbohydrate (31.6% vs. 42.4%), protein (13% vs. 16.4%), and fat (33.6% vs. 35.7%) intakes compared to non-AUD group. This is in accordance with previous observations that energy derived from alcohol might replace the energy from macronutrients [23]. The biochemical mechanisms involved in abdominal adiposity from heavy alcohol use are not clear, although heavy alcohol use seems to suppress fat oxidation, resulting in increased lipid storage [24].

Hormones may also be involved in abdominal adiposity in AUD individuals. HPA-axis activation by alcohol results in increased release of cortisol, which is associated with changes in fat distribution. Specifically, fat deposition in visceral adipose tissue is promoted by cortisol binding to glucocorticoid receptors, and visceral adipose tissue has a high density of these receptors [25]. Epinephrine and norepinephrine, along with cortisol, are hormones released during the stress response by the adrenal gland. Chronic alcohol use also results in a hormonal imbalance with a persistent increase in stress hormones [26]. Studies on the association of HPA axis activation with alcohol intake have provided mixed results regarding increased epinephrine and norepinephrine concentrations during alcohol withdrawal [27,28,29], cortisol concentrations during withdrawal [30,31], and cortisol concentrations [32] in binge drinkers. In this study, the AUD group had increased epinephrine and norepinephrine concentrations compared to the non-AUD group; however, cortisol concentrations were not different. It must be noted that participants were current drinkers, and some of them were binge drinkers. Differences in the years of having AUD, and age between the participants in this work and previous studies make it difficult to make conclusions.

Changes in the endogenous opioid system (physiological increases in plasma beta-endorphin concentrations under stress conditions) may play a role in some metabolic alterations associated with obesity, including hyperglycaemia and hyperinsulinemia [33,34]. Although acute exposure to alcohol may cause a rapid and short-term increased release of βeta-endorphin by the pituitary and hypothalamus [35,36] that is related to the dose [37], long-term alcohol abuse may result in decreased release of β-Ε and decreased density and activation of opioid receptors [6]. Therefore, it is unlikely that an association between beta-endorphin and abdominal adiposity in AUD individuals exists. In addition, beta-endorphin may play a role in the pleasure derived from the consumption of dietary fat (hedonic preference), as indicated by the neuronal activation in beta-endorphin neurons followed by dietary fat ingestion [38,39]. Beta-endorphin was decreased in the AUD group, whereas dietary fat intake was similar, which also supports a lack of an association.

Malnutrition in individuals with AUD is a common finding in the research literature [40]. The HPA-axis may be involved in nutrient deficiencies, and may be associated with metabolic alterations in these individuals [11]. Even though AUD group was overweight, malnutrition could also exist. Indeed, malnutrition (insufficient supply or incorrect absorption of essential nutrients) is associated not only with decreased BMI, but also with obesity [41]. Incorrect dietary choices in AUD individuals are not uncommon [12]. In the AUD group, thiamine and folic acid intake was lower when compared to the non-AUD group; however, both groups had sufficient thiamine and insufficient folic acid intake (according to RDA). The relation of alcohol intake to folic acid is well studied and folic acid deficiency can lead to serious clinical consequences [42]; it should therefore be taken into consideration.

Regarding the correlation between HPA-axis activity, nutrient intake, and body composition, only a negative association between Mg and caffeine in the AUD group was found. In the non-AUD group, the only findings were a positive correlation between epinephrine and B12, and a negative association between cortisol and vitamin E. The interpretation of these results is difficult and further investigation is needed; the the sample size was quite small, which is a main limitation of this study. Another limitation is the exclusion of female participants, who may respond differently to chronic alcohol abuse.

## 5. Conclusions

This was a first attempt to determine whether a correlation between HPA-axis activity and nutritional status in individuals with AUD exists. Most likely, excessive alcohol intake was the main cause of abdominal adiposity found in the participants with AUD. Although these individuals exhibited low concentrations of plasma beta-endorphin, no clear correlation with nutritional status was evident. Nevertheless, the results of this study provide a further confirmation that AUD individuals make unhealthy dietary choices that may be associated with metabolic alterations. Therefore, it is suggested that they should be periodically screened for malnutrition, and particularly for folic acid deficiency. Finally, more research with larger sample sizes should be conducted to elucidate this possible correlation between HPA-axis activation and nutritional status in individuals with AUD, and should take into consideration participants’ characteristics such as gender, genetic predisposition to alcoholism, and years of having AUD. Since body weight can be either increased or decreased when malnutrition occurs, especially in individuals with alcohol or other substance use disorder, more components of nutritional assessment, such as biochemical and dietary analyses, should be routinely used in this population.

## Figures and Tables

**Table 1:** Characteristics of the AUD (n = 14) and non-AUD (n = 14) group.

Variable	AUD Group	Non-AUD Group
Age (yrs)	33.7 ± 13.3	34.1 ± 9.7
IPAQ (MET-min/week)	1057.6 ± 705.8	1267.4 ± 376.5
AUDIT score	16.8 ± 4.9	2.2 ± 1.6
Standard drinks * per week	17.9 ± 9.6	2.9 ± 3.0
Cigarettes per day	11.5 ± 8.7	11.4 ± 10.3

AUD: Alcohol Use Disorder; IPAQ: International Physical Activity Questionnaire; MET: metabolic equivalent of task; AUDIT: Alcohol Use Disorders Identification Test; * A standard drink equivalent is 14 g of alcohol, e.g., 150 mL of wine (containing 12% alcohol), 350 mL of beer (containing 5% alcohol).

**Table 2:** Anthropometric and body composition characteristics of AUD versus non-AUD group.

Index	Group	N	Mean Rank	U	Z	*p*
BMI	AUD Non-AUD	14 14	14.86 14.14	93.000	−0.230	>0.05
WC (cm)	AUD Non-AUD	14 14	18.64 10.36	40.000	−2.666	<0.01
WHR	AUD Non-AUD	14 14	17.61 11.39	54.500	−2.006	<0.05
BF (%)	AUD Non-AUD	14 14	13.68 15.32	86.500	−0.528	>0.05

BMI: body mass index; BF: body fat; WC: waist circumference; WHR: waist to hip ratio.

**Table 3:** Nutrient intake of AUD versus non-AUD group.

Index	Group	N	Mean Rank	U	Z	*p*
Energy (kcal)	AUD Non-AUD	14 14	14.21 14.79	94.000	−0.184	>0.05
Carbohydrates (g)	AUD Non-AUD	14 14	11.79 17.21	60.000	−1.746	>0.05
Protein (g)	AUD Non-AUD	14 14	12.86 16.14	75.000	−1.057	>0.05
Total fat (g)	AUD Non-AUD	14 14	15.21 13.79	88.000	−0.460	>0.05
Saturated fatty acids (g)	AUD Non-AUD	14 14	13.14 15.86	79.000	−0.873	>0.05
Monounsaturated fatty acids (g)	AUD Non-AUD	14 14	16.57 12.43	69.000	−1.333	>0.05
Polyunsaturated fatty acids (g)	AUD Non-AUD	14 14	11.79 17.21	60.000	−1.746	>0.05
Cholesterol (mg)	AUD Non-AUD	14 14	14.71 14.29	95.000	−0.138	>0.05
Fibre (g)	AUD Non-AUD	14 14	12.00 17.00	63.000	−1.608	>0.05
Alcohol (g)	AUD Non-AUD	14 14	21.07 7.93	6.000	−4.231	<0.001
Caffeine (mg)	AUD Non-AUD	14 14	16.07 12.93	76.000	−1.011	>0.05
Calcium (Ca)	AUD Non-AUD	14 14	15.79 13.21	80.000	−0.827	>0.05
Copper (Cu)	AUD Non-AUD	14 14	12.04 16.96	63.500	−1.586	>0.05
Iron (Fe)	AUD Non-AUD	14 14	10.36 18.64	40.000	−2.666	<0.01
Magnesium (Mg)	AUD Non-AUD	14 14	13.93 15.07	90.000	−0.368	>0.05
Manganese (Mn)	AUD Non-AUD	14 14	13.57 15.43	85.000	−0.550	>0.05
Phosphorus (P)	AUD Non-AUD	14 14	14.43 14.57	97.3000	−0.046	>0.05
Potassium (K)	AUD Non-AUD	14 14	13.93 15.07	90.000	−0.368	>0.05
Selenium (Se)	AUD Non-AUD	14 14	12.79 16.21	74.000	−1.103	>0.05
Sodium (Na)	AUD Non-AUD	14 14	12.43 16.57	69.000	−1.332	>0.05
Zinc (Zn)	AUD Non-AUD	14 14	14.93 14.07	92.000	−0.276	>0.05
Vitamin A (IU)	AUD Non-AUD	14 14	12.71 16.29	73.000	−1.149	>0.05
Vitamin A (RE)	AUD Non-AUD	14 14	12.21 16.79	66.000	−1.471	>0.05
Thiamine	AUD Non-AUD	14 14	10.86 18.14	47.000	−2.344	<0.05
Riboflavin	AUD Non-AUD	14 14	12.18 16.82	65.500	−1.495	>0.05
Niacin	AUD Non-AUD	14 14	13.36 15.64	82.000	−0.736	>0.05
Pantothenic acid	AUD Non-AUD	14 14	14.36 14.64	96.000	−0.092	>0.05
Vitamin B6	AUD Non-AUD	14 14	13.11 15.89	78.500	−0.898	>0.05
Folic acid	AUD Non-AUD	14 14	10.86 18.14	47.000	−2.343	<0.05
Vitamin B12	AUD Non-AUD	14 14	14.36 14.64	96.000	−0.092	>0.05
Vitamin C	AUD Non-AUD	14 14	13.00 16.00	77.000	−0.965	>0.05
Vitamin D	AUD Non-AUD	14 14	12.50 16.50	70.000	−1.287	>0.05
Vitamin E	AUD Non-AUD	14 14	12.89 16.11	75.500	−1.034	>0.05
Vitamin K	AUD Non-AUD	14 14	14.43 14.57	97.000	−0.046	>0.05

**Table 4:** Parameters of HPA-axis of AUD versus non-AUD group.

Index	Group	N	Mean Rank	U	Z	*p*
**Beta-Endorphin**	AUD Non-AUD	14 14	8.64 20.36	16.000	−3.771	**<0.001**
**ACTH**	AUD Non-AUD	14 14	15.57 13.43	83.000	−0.690	>0.05
**Cortisol**	AUD Non-AUD	14 14	12.64 16.36	72.500	−1.195	>0.05
**Epinephrine**	AUD Non-AUD	14 14	17.79 11.21	52.000	−2.120	**<0.05**
**Norepinephrine**	AUD Non-AUD	14 14	18.46 10.54	42.500	−2.551	**<0.05**
**Dopamine**	AUD Non-AUD	14 14	16.00 13.00	77.000	−0.967	>0.05

ACTH: Adrenocorticotropin hormone.

## Data Availability

The data that support the findings of this study are available from the corresponding author, [author initials], upon reasonable request.
